# The Ligature and Value of Dry Sterilised Catgut1Author’s abstract of a paper read at Louisville, Ky., September 20, 1900, before the American Association of Obstetricians and Gynecologists.

**Published:** 1900-11

**Authors:** J. H. Carstens

**Affiliations:** Detroit, Mich.


### Abstracts.

### THE LIGATURE AND VALUE OF DRY STERILISED CATGUT.[1]

By J. H. CARSTENS, M. D., Detroit, Mich.

FINALLY, the profession found that silk was the best for that purpose, and so for the first half of the 19th century virtually only silk was used. During the last quarter of that century, with the advent of abdominal surgery, requiring ligatures to be buried and beyond reach after the external wound had healed, the trouble began. Silk was a foreign body and would often cause fistulæ, and the profession looked around for other material, and even used

1. Author's abstract of a paper read at Louisville, Ky., September 20, 1900, before the American Association of Obstetricians and Gynecologists.

various metals, but they were generally found wanting. Then animal ligatures of various kinds were tried. The greatest trouble with these has been to make them absolutely sterile. Every kind of animal tissue from the tail of the kangaroo to the fascia lata of the deer has been used and again dropped.

I have tried the different kinds of catgut ligatures, koumoled, formaldehyded, chromocised, and always found something wrong. The great objection that I had to all these ligatures was that they *last too long*.

It really seems too absurd to me, when I hear men talking about using twenty, thirty or forty-day catgut, as though it made any difference whether it lasted ten days, twenty days or two months. If the parts are not *healed* in a few days or a week, they will not heal in a month, if you simply hold them in apposition with the ligature. When I have taken out pieces of ligatures weeks and months after being put in place, I have become perfectly disgusted with the various sutures.

Still, catgut is just the ideal ligature that we want in abdominal surgery. Again and again I have approached the subject and experimented, but have often become discouraged. Reverdin, Docderlein and others, recommended dry sterilised catgut, but no good apparatus could be had until I heard some years ago about the dry sterilised catgut, as prepared by Boerckmann. The only thing that I did not like was to use oil paper or paraffin paper for the purpose of, in a way, oiling the ligature. That is just what I did not want in a ligature. What I wanted is a *plain, pure animal fiber*, as small as possible or as fine and as light as possible, to hold the parts in apposition for a few days or to control a bloodvessel.

After I had sterilised some, I was astonished how strong the catgut was after being subject to an intense heat. First using some pretty heavy, then I began to use it lighter and lighter and finally got down to No. 3 of the finest German catgut. (Ten strands of ten feet each in a box, is the way I got it.) This I used for tying the pedicle and bloodvessels, and it is more than strong enough for that purpose. For fine work, that is intestinal surgery or to sew the peritoneum together, I use only No. 1 or No. 0. For instance, in operating for appendicitis, that is amply sufficient in strength, as agglutination of the peritoneum takes place so rapidly.

I prepare these ligatures myself (although they are now in the market) in the following manner: The catgut is put in ether for a few days or a week until the fat is all removed and then cut in strips of 18 or 20 inches long. Three of these are wrapped in fine tissue paper. This is then placed in a small envelop, the latter closed, then placed in the Boerckmann steriliser and subject to dry heat for three hours. The thermometer is kept in the apparatus, so that you can see that the heat is at least 300 degrees. At the expiration of that time the heat is shut off and the ligatures remain in the apparatus without disturbance for twelve to eighteen hours, which gives any spores that may be present an opportunity to develop. Then the heat is again used and the ligatures are subject to another 300 degrees.

No contamination can take place while they are in the envelop, unless the envelop becomes moist. I have had a package in my satchel which I carried all over Europe, and whenever anybody wanted to try it, I gave them a couple of envelops. The envelops were loose, came in contact with all kinds of things, but were kept dry, and when I returned I made bateriological tests and found that the catgut was still absolutely sterile.

I have repeatedly tested this catgut and always found it absolutely sterile.

The points I want to make about the dry sterilised catgut ligatures are the following:

1. All buried sutures ought to be absorbable.
2. All absorbable ligatures must be absolutely sterile.
3. Chemicalised sutures are no more sterile than plain sutures.
4. A suture that is chemicalised is harder and remains longer in the tissues.
5. This latter is no advantage but a disadvantage. If in a special case it is desirable that a suture should remain longer, dry sterilised kangaroo tendon can be used.

